# Olfactory marker protein interacts with adenosine nucleotide derivatives

**DOI:** 10.1016/j.bbrep.2020.100887

**Published:** 2021-01-12

**Authors:** Noriyuki Nakashima, Kie Nakashima, Akiko Nakashima, Makoto Takano

**Affiliations:** a Department of Physiology, Kurume University School of Medicine, 67 Asahi-machi, Kurume, Fukuoka, 830-0011, Japan; b Laboratory of Developmental Neurobiology, Graduate School of Biostudies, Kyoto University, Yoshida Hon-machi, Kyoto, 606-8501, Japan

**Keywords:** Olfactory marker protein, cAMP-binding protein, Adenosine nucleotide derivatives, Bioluminescent resonant energy transfer

## Abstract

Olfactory marker protein (OMP) is a genetic signature for mature olfactory receptor neurons (ORNs). Recently, it has been proposed that OMP directly captures odour-induced cAMP to swiftly terminate the olfactory signal transduction to maintain neuronal sensitivity. In the present study, we show that OMP can also interact with other adenosine nucleotides as ATP, ADP and AMP with different affinities. We performed bioluminescent resonant energy transfer (BRET) assay to measure the binding actions of the adenosine nucleotide derivatives in competition to cAMP. Amongst all, ATP showed the bell-shape affinity to OMP in the presence of cAMP; ADP and AMP showed fewer affinities to OMP than ATP. In the absence of cAMP analogues, ATP alone bound to OMP in a dose dependent manner with a lower affinity than to cAMP. Thus, OMP possessed different affinities to ATP in the presence or absence of cAMP. OMP may interact differentially with ATP and cAMP depending on its supply and demand along the cAMP-associated signalling in the limited spaces of cilia of ORNs.

## Highlights

• Olfactory marker protein (OMP) contains cAMP-binding sites.
• The affinity of OMP towards adenosine nucleotide derivatives was studied.
• OMP showed sigmoid-shaped affinity towards ATP.
• OMP showed U-shaped affinity towards ATP in competition with cAMP.
• OMP dose-dependently and differentially captured ATP.

## 1 Introduction

Olfaction starts with olfactory receptor neurons (ORNs), utilizing cAMP as a second messenger [1]. Odorant receptors are polymodal sensors for chemical and mechanical stimuli that share the cAMP-associated signalling pathway [[1], [2], [3], [4]]. Thus, ORNs incessantly and resiliently utilize cAMP for responding to external stimuli.

These investigations in olfaction have been facilitated by the discovery of olfactory marker protein, (OMP), which genetically labels mature ORNs [[5], [6], [7], [8]]. Close examinations have revealed that knocking-out of the OMP gene reduces odour discrimination ability in mice [[9], [10], [11], [12], [13], [14]], prolongs odour response kinetics mediated by the Ca^2+^-permeable cyclic nucleotide-gated A2 (CNGA2) channels [[15], [16], [17], [18]], and delays the Ca^2+^ extrusion via the Na^+^-Ca^2+^ exchanger (NCX) in ORNs [19]. Furthermore, OMP-KO delays the maturation of axonal projections from ORNs to the olfactory bulb during development [15,20,21], in which basal cAMP levels play a key role [17,18,[22], [23], [24], [25]].

By searching through the amino acid sequence of OMP, we realized that OMP contains a cyclic nucleotide binding domain (CNBD)-like motif [17]. OMP was a cAMP binding protein in ORNs and regulated phasic and tonic cAMP signalling kinetics [[15], [16], [17],[25], [26], [27]]. Previously, we assessed the direct physical interaction between OMP and cAMP *in vitro* by performing bioluminescence resonance energy transfer (BRET) experiments and determined the apparent affinities to cAMP [17].

On the other hand, OMP is known to interact with transcription factors [28,29] and seems to affect cell proliferation [30]. In general, ATP plays a pivotal role in cell cycle regulations. Therefore, we hypothesized that OMP would be also essential in balancing the ATP homeostasis. Therefore, we examined the affinity towards other derivatives of adenosine-related nucleotides including ATP, ADP and AMP by *in silico* simulation and BRET analysis.

## 2 Materials and methods

### 2.1 Ligand-binding computer simulation

We consulted the Protein Data Bank (PDB) for a solution structure of OMP determined by nuclear magnetic resonance (Model 1 from a 1ZRI file) [17,31]. The OMP data were further modified by adding polar hydrogen atoms and rendered into an analysis grid using AutoDockTools under a Python platform [32]. Structural data for ATP (CID 5957), ADP (CID 6022) and AMP (CID 6083) were obtained from the NCBI PubChem Database in a 3D-structure-data file format, which was then converted into PDB format using OpenBabel [33]. Docking simulations were performed using AutoDockVina [34] (The Scripps Research Institute, CA, USA). The results were analyzed with the Python Molecular Viewer [32], including molecular surface rendering and rendering into PNG image files. The PDB data were modified by removing water molecules. Selenomethionine (MSE in PDB data) was not edited because the N-terminus of OMP was located at the edge of the globular structure.

### 2.2 Generation of cDNA constructs

We used PfuUltra Fusion DNA polymerase (Agilent, CA, USA) and Q5 High-Fidelity DNA polymerase (New England Biolabs, MA, USA) for PCR [17]. We used the pCI mammalian expression vector (Promega, WI, USA) for exogenous expression in HEK293T cells (ATCC, VA, USA). The cDNAs for mouse OMP (NM_011010) and Renilla luciferase (Rluc) were amplified from pGL4.74 [hRluc/TK] (Promega, WI, USA) via PCR and then inserted into the pCI vector. Rluc lacking the stop codon was inserted immediately upstream of OMP in frame with a linker sequences corresponding to valine-glutamine-phenylalanine-phenylalanine.

### 2.3 Heterologous cDNA expression

HEK293T cells were cultured in Dulbecco's MEM (DMEM; Wako Pure Chemical, Osaka, Japan) supplemented with 10% foetal bovine serum (FBS: Sigma-Aldrich, MO, USA) without antibiotics at 37 °C in 5% CO_2_. The plasmids containing cDNAs for Rluc-OMP fusion genes or Rluc (5 μg) were transfected into HEK293T cells using Effectene Transfection Reagent (QIAGEN, Hilden, Germany) following the manufacturer's protocol as previously described [17].

### 2.4 Optical spectrum analysis for BRET

The transfected HEK293T cells were incubated at 37 °C with 5% CO_2_ for 24 h, then collected via centrifugation at 2000 rpm for 5 min, and the supernatant was discarded. The cell pellet was resuspended in divalent cation-free phosphate buffered saline (PBS(−):100 μL) and disrupted by sonication at 3.1 kHz for 5 min in an ice-cold water bath. The lysate was centrifuged to remove the cell debris. The supernatant containing the proteins was further rinsed using Vivaspin ultrafiltration columns (3 kDa: Sartorius Stedim Biotech, Göttingen, Germany) and then resuspended in 100 μL PBS(−) for the following experiments. For BRET analysis, the 1-mL test solution was prepared in divalent cation-free PBS (approximately a 1000-fold dilution of the original lysate to yield calibrated BRET signals greater than 10^5^ in arbitrary units) and supplemented with 8-(2-[7-Nitro-4-benzofurazanyl]aminoethylthio)adenosine-3′,5′-cyclic monophosphate (8NBD-cAMP; BIOLOG Life Science Institute, Bremen, Germany) in a series of dilutions (200 nM - 10 μM) or 2′,3′-O-Trinitrophenyl-adenosine-5′-triphosphate (TNP-ATP; Wako Pure Chemical, Osaka, Japan) in a series of dilutions (10 nM - 100 μM). The test tubes were placed in a block thermostat maintained at 24 °C, and the thermostat block was completely covered with C-mount laser housing attached to the detecting fibre optics. The experiments were performed in a self-built darkroom. The signals were detected through fibre optics, relayed to a spectrometer (Optics 250is, Bruker K.K., USA), enhanced with an image intensifier (M7971-81, Hamamatsu Photonics, Shizuoka, Japan), captured with an infrared digital CCD camera (ORCA-R2, Hamamatsu photonics, Shizuoka, Japan), and recorded at 5-sec intervals with 10-fold gain. We confirmed that 8NBD-cAMP had no autoluminescence in the absence of Rluc or in the presence of Rluc alone without its substrate [17]. After background calibration, 1 μL of Rluc substrate (coelenterazine: Stop & Glo, Promega, WI, USA) was applied quickly, and the emitted light at wavelengths 350 to 650 nm was detected in the same manner. The acceptor/donor ratios were determined by the ratios of signal intensities at 536 nm and 480 nm, and compared in the presence and absence of 8NBD-cAMP to yield BRET ratios as described previously [17,35]. The BRET ratio for nonspecific luminescence by Rluc alone was further subtracted from the BRET ratio of Rluc-OMP to correct for background noise offline. At the condition where OMP was presaturated with 5 μM of 8NBD-cAMP, the adenosine nucleotide analogues (ATP, ADP or AMP, were added as competitors at different concentrations to yield the shift of the BRET ratios (ΔBRET). The spectrum for each recording was the average over 5 sessions from 3 samples. No PDE inhibitor, AC inhibitor or proteinase inhibitor cocktail were added to the OMP-containing solutions because these molecules might also interact with OMP as adenosine nucleotide analogues.

## 3 Results

### 3.1 Docking simulation between OMP and adenosine nucleotide derivatives

**Fig. 1:**
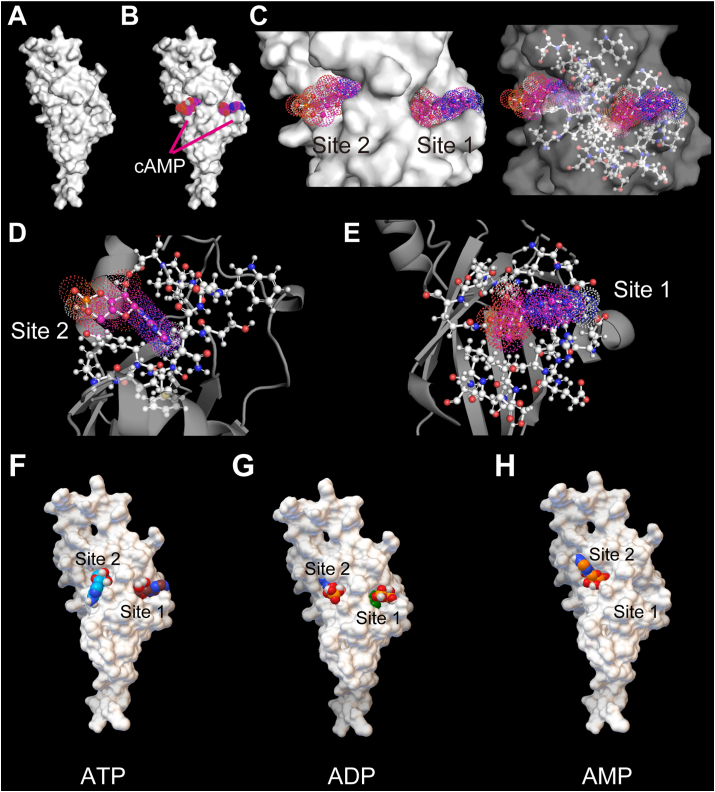
Simulated conformation of binding between OMP and ATP derivatives. (**A**) The crystal structure of OMP shown with the solvent-accessible surface area (SASA). (**B**) The conformation of OMP and two cAMP molecules. The cAMP molecules are shown with the coloured dotted surface. (**C**) Close-up views of cAMP-binding pockets in OMP; Sites 1 and 2 (left panel) with the solid SASA; and the ball-and-stick model of pockets-forming amino acids with the transparent SASA (right). The cAMP molecules are shown with coloured dotted surface and the ball-and-stick structures underneath. (**D,E**) Close-up views of (**D**) Site 2 and (**E**) Site 1. The orifices of Sites 1 and 2 are shown in ball-and-stick models, and the other parts of OMP are distinguished in ribbon models. (**F–H**) The binding conformations of OMP with (**F**) ATP, (**G**) ADP or (**H**) AMP, respectively. ATP, ADP and AMP are shown in the solid surface models.

OMP is a globular protein (Fig. 1A) [5,8]. Previously, we detected two major pockets in OMP, namely Sites 1 and 2; these pockets were proposed to directly bind to cAMP (Fig. 1B and C) [17]. Site 1 was a pocket containing a classical cAMP-binding motif, and Site 2 was another pocket detected by *in silico* docking simulation (Fig. 1D and E; Movies S1 and S2) [17]. First, we consulted a public databank to obtain the data for 3D structures of OMP, ATP, ADP and AMP. Docking simulations, performed in a desktop computer, predicted that OMP might bind to ATP, ADP and AMP with the identical pockets for cAMP-binding (Fig. 1F–H) [17] with simulated free energy of binding (ΔG) as follows: ΔG_ATP_, −29.8 and −24.6; ΔG_ADP_, −25.9 and −25.5; and ΔG_AMP_, not simulated and −23.4; the highest simulated values in kJ/mol for Site 1 and 2, respectively.

Supplementary data related to this article can be found at https://doi.org/10.1016/j.bbrep.2020.100887.

The following are the supplementary data related to this article:

Movie S1 **Docking conformation of OMP with cAMP at Sites 1 and 2.** Sites 1 and 2 of OMP were shown in the ball-and-stick model with carbon atoms in grey; the rest of OMP structure was shown in the ribbon model. The cAMP molecule was shown in the ball-and-stick-model with carbon atoms in pink with the coloured dotted surface. Movie S1

Movie S2 **Docking conformation of OMP with cAMP at Sites 1 and 2.** The transparent solvent-accessible surface area was added to the OMP structure in Movie S1. Movie S2

**Fig. 2:**
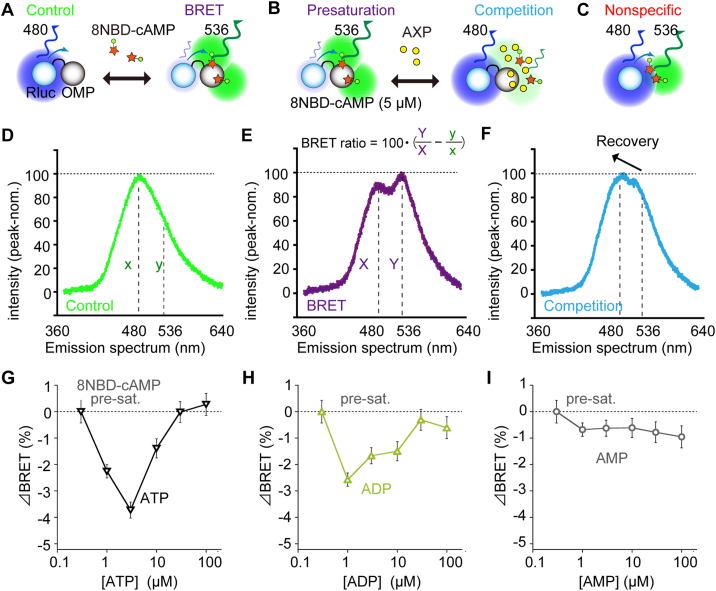
ATP and ADP showed competitive interaction with cAMP bound to OMP. (**A**) Scheme of BRET measurement using Rluc-OMP fusion protein and 8NBD-cAMP. (**B**) Scheme of competition assay from a condition presaturated with 5 μM of 8NBD-cAMP. AXP indicates ATP, ADP or AMP. (**C**) Scheme of nonspecific BRET occurred between Rluc alone and 8NBD-cAMP. (**D, E**) Representative emission spectra of OMP-Rluc (**D**) without or (**E**) with 8NBD-cAMP (Control). The BRET ratio was defined as the equation in (**E**): X, Y, x and y are indicated in (**D**) and (**E**). Nonspecific BRET was subtracted offline. (**F**) Representative emission spectra of Rluc-OMP with additional competitive ATP with the recovery peak -shift, which resulted in the decrease in the BRET ratio (ΔBRET). (**G-I**) ΔBRET from the presaturated condition (pre-sat.) by adding (**G**) ATP, (**H**) ADP or (**I**) AMP. *n* = 3 each experiments; each experiment was averaged over 10 samplings at each concentration. Peak-norm.; normalized to the peak value. Mean ± s.e.m.

The competitive assay in this report was based on the previous study [17], in which we assessed the direct physical interaction between OMP and fluorescently labelled 8NBD-cAMP by BRET experiments (Fig. 2A–C). In the BRET experiments, luminescence energy from luciferase is transferred in a restricted manner only to the nearby fluorescent molecules that are in very close proximity and positionally well-oriented [35]. In this study, Rluc (energy donor: emission peak at 480 nm) was fused to OMP (Rluc-OMP) such that BRET would occur upon close access of fluorescent 8NBD-cAMP (energy acceptor; emission peak at 536 nm) to luminescent Rluc-OMP as previously reported [17] (Fig. 2A,D,E). The relative shift of emission peak from 480 to 536 nm was evaluated as the BRET ratio (Fig. 2E) [17]. The higher concentration of 8NBD-cAMP would result in the nonspecific background BRET signals (Fig. 2C), which were subtracted offline. We determined that BRET ratio between Rluc-OMP and 8NBD-cAMP was saturated by 5 μM fluorescently labelled-cAMP [17]. Setting this concentration as a saturation standard hereafter (presaturation; Fig. 2B), we performed the subsequent competitive assay for ATP, ADP and AMP (competition; Fig. 2B,F–I).

### 3.2 ATP and ADP competed with cAMP in a U-shaped manner

**Fig. 3:**
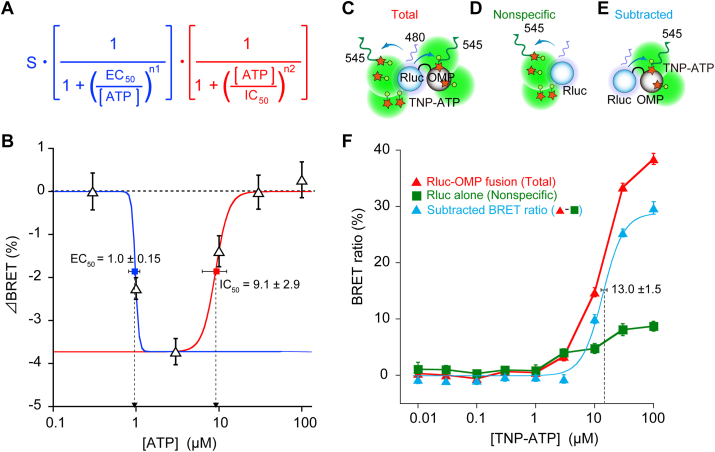
OMP interacts with ATP differently depending on the presence of cAMP. (**A**) Hill equation for the ATP-cAMP competitive assay in Fig. 2C. Two reactions of ATP for positive competition with cAMP (EC_50_, blue) and for negative competition at high concentrations (IC_50_, red) are assumed. Hill coefficients are n1 and n2. S, maximal decrease of ΔBRET signals. (**B**) ΔBRET for ATP fitted with the equation in (**A**); *n* = 3 each experiments. S = −3.7 ± 0.3%. For positive competition at low concentrations of ATP with EC_50_, blue (affinities to OMP; ATP > cAMP); for negative competition at high concentrations of ATP with IC_50_, red (affinities to OMP; ATP < cAMP). The Hill coefficients were approximately 1 (n1 = 1.0 ± 0.2; and n2 = 1.2 ± 0.1), suggesting that the ATP interaction with OMP/cAMP is simple competition for both reactions. (**C-E**) Scheme of (**C**) Rluc-OMP or (**D**) Rluc alone interacting with TNP-ATP (peak at 545 nm) causing BRET with nonspecific background signals and (**E**) subtracted true signals. (**F**) The actual BRET ratio (blue-filled triangle) was determined by subtracting the BRET ratios of Rluc-OMP (total signals; red-filled triangle; *n* = 6) and Rluc (nonspecific signals; green-filled square; *n* = 10). Mean ± s.e.m. . (For interpretation of the references to colour in this figure legend, the reader is referred to the Web version of this article.)

By addition of adenosine nucleotide derivatives to Rluc-OMP presaturated with 8NBD-cAMP, BRET spectrum showed the peak-recovery at 480 nm with the diminution at 536 nm by competition between fluorescent cAMP and non-fluorescent adenosine nucleotide derivatives (Fig. 2B). This shift resulted in the decrease in BRET, which was then defined as ΔBRET (Fig. 3B–E). Both ATP and ADP showed U-shaped competitive effects on 8NBD-cAMP that peaked at 1–3 μM, implying an auto-inhibitory effect on binding to OMP at higher ATP or ADP concentrations (Fig. 2G and H), whereas AMP showed the least competitive effects on 8NBD-cAMP (Fig. 2I).

### 3.3 ATP alone interacted with OMP in a dose-dependent manner

We further investigated the affinity of ATP in details, which is abundant in the cytoplasm. The U-shaped plot for ATP was fitted with two sigmoidal curves assuming positive and negative actions as a competitor in parallel, which resulted in an EC_50_ and IC_50_ of 1.0 ± 0.15 and 9.1 ± 2.9 μM, respectively (Fig. 3A and B). Considering that OMP is a structurally flexible protein [35,36] the presaturation with cAMP might change the affinities between OMP and other adenosine nucleotides. We then performed a binding assay between fluorescent TNP-ATP and Rluc-OMP in the absence of 8NBD-cAMP (Fig. 3C–E). BRET occurred only at relatively high concentrations of TNP-ATP, with an EC_50_ of 13.0 ± 1.5 μM (Fig. 3F). These results imply that the affinities of ATP to OMP could be enhanced in the presence of cAMP.

## 4 Discussion

We identified that ATP or ADP also bound to OMP and the affinities of ATP were dependent on the presence of cAMP. In general, the intracellular ATP is present in the millimolar range [37]. The results indicate that OMP should be saturated with cytosolic ATP in the absence of cAMP. However, at micromolar concentrations, ATP was less strongly bound to OMP than cAMP. Considering that the cilia of ORNs is the limited space separated from the mitochondria situated just beneath the ciliary spaces [1,38], the local ATP concentrations in the cilia could be below the concentrations in the cytosol due to the diffusion restriction or local hydrolysis, while ATP should be promptly provided to produce cAMP incessantly. Therefore, the evoked cAMP increase under sensory stimulation might compete with the ATP bound to OMP; ATP can be released instead as an alternative source of ATP and absorber of cAMP simultaneously in the cilia of ORNs. On the other hand, at the extremely low concentrations of ATP, OMP might maintain binding to ATP instead of capturing the produced cAMP, which may prolong the actions of free cAMP without consuming further ATP. It should be also noted that ATP also exists in the form of MgATP, and its availability can be further modulated by OMP [37]. Although the mechanisms by which ATP or ADP at low concentration (1–3 μM) competed with 8NBD-cAMP remain unknown, cross-talk among various nucleotides may occur in the vicinity of OMP including cGMP [17] or GTP. OMP might contribute to the simultaneous regulation of different species of nucleotides by directly binding and releasing them as a general nucleotide buffer.

Although OMP-KO is not a lethal phenotype in the laboratory, evolutionary invariance at the cAMP-binding sites substantiates the necessity of OMP for survival in the wild [17,23,26,27,39]. In addition to the temporal aspects of cAMP signalling, the cell is functionally compartmentalized by various cAMP-binding proteins into specific subcellular domains [40,41], and the actions of cAMP are more diversified than a traditional robust binary switch [42]. We did not consider the secondary conformational changes resulting from the consecutive nucleotide binding to OMP in the present study. However, OMP itself is structurally flexible [31,36] and is dimerized in certain situations [29]. Thus, Sites 1 and 2 might serve as an allosteric cAMP-binding site for precise switching of the conformation and function of OMP. Moreover, OMP reportedly enters the nucleus to interact with transcription factors [28] and affects cell proliferation by unknown mechanisms [30]. Given that OMP can capture various nucleotides using its binding pockets (Sites 1 and 2), OMP may shuttle nucleotides from one compartment to another and exchange them at a distance.

Besides the olfactory system, extra-olfactory OMP-expressing tissues have been found in the peripheral sensory and central nervous systems, as well as in the uroreproductive systems [[43], [44], [45], [46], [47]], and these tissues are potentially capable of life-long renewal and regeneration [[48], [49], [50], [51], [52], [53], [54], [55], [56], [57], [58]]. OMP can regulate the cAMP-associated signalling and metabolism, operating as a cAMP reservoir [27]. Notably, OMP was expressed in a part of cells in the hypothalamus [7], where OMP's interaction partner, Brain Expressed/X-Linked Protein (BEX), is expressed [28,29,59]. The maintenance of cell renewal in hypothalamus controls aging [60], and the hypothalamic projection regulates the on-demand neurogenesis in the subventricular zone in response to changes in physiological and environmental states [61], therefore the stable hypothalamic neurogenesis would be essential in balancing the homeostasis in the long term. Collectively, OMP might provide a pivotal platform for regulating intracellular nucleotide signalling and metabolism during cell maturation and regeneration in various parts of the body.

## Credit author statement

Noriyuki Nakashima: Conceptualization, Investigation, Formal analysis, Supervision, Project administration, Writing - Reviewing and Editing original and revised manuscripts and Funding acquisition. Kie Nakashima: Conceptualization, Investigation, Validation and Resource, Methodology, Formal analysis, Writing - Original Draft. Akiko Nakashima: Investigation by simulation, Methodology, Formal analysis, Writing - Original Draft and Funding acquisition. Makoto Takano: Writing - Reviewing and Editing original and revised manuscripts, and Funding acquisition.

## Declaration of competing interest

Authors declare no conflicts of interests.

## References

[1] Takeuchi H., Kurahashi T. (2002). Photolysis of caged cyclic AMP in the ciliary cytoplasm of the newt olfactory receptor cell. J. Physiol..

[2] Chen X., Xia Z., Storm D.R. (2012). Stimulation of electro-olfactogram responses in the main olfactory epithelia by airflow depends on the type 3 adenylyl cyclase. J. Neurosci..

[3] Connelly T., Yu Y., Grosmaitre X., Wang J., Santarelli L.C., Savigner A., Qiao X., Wang Z., Storm D.R., Ma M. (2015). G protein-coupled odorant receptors underlie mechanosensitivity in mammalian olfactory sensory neurons. Proc. Natl. Acad. Sci. U.S.A..

[4] Grosmaitre X., Santarelli L.C., Tan J., Luo M., Ma M. (2007). Dual functions of mammalian olfactory sensory neurons as odor detectors and mechanical sensors. Nat. Neurosci..

[5] Danciger E., Mettling C., Vidal M., Morris R., Margolis F. (1989). Olfactory marker protein gene: its structure and olfactory neuron-specific expression in transgenic mice. Proc. Natl. Acad. Sci. U.S.A..

[6] L Margolis F. (1972). A brain protein unique to the olfactory bulb. Proc. Natl. Acad. Sci. U.S.A..

[7] Monti-Graziadei G.A., Margolis F.L., Harding J.W., Graziadei P.P. (1977). Immunocytochemistry of the olfactory marker protein. J. Histochem. Cytochem..

[8] Potter S.M., Zheng C., Koos D.S., Feinstein P., Fraser S.E., Mombaerts P. (2001). Structure and emergence of specific olfactory glomeruli in the mouse. J. Neurosci..

[9] Buiakova O.I., Baker H., Scott J.W., Farbman A., Kream R., Grillo M., Franzen L., Richman M., Davis L.M., Abbondanzo S., Stewart C.L., Margolis F.L. (1996). Olfactory marker protein (OMP) gene deletion causes altered physiological activity of olfactory sensory neurons. Proc. Natl. Acad. Sci. U.S.A..

[10] Ivic L., Pyrski M.M., Margolis J.W., Richards L.J., Firestein S., Margolis F.L. (2000). Adenoviral vector-mediated rescue of the OMP-null phenotype *in vivo*. Nat. Neurosci..

[11] Kass M.D., Moberly A.H., McGann J.P. (2013). Spatiotemporal alterations in primary odorant representations in olfactory marker protein knockout mice. PloS One.

[12] Lee A.C., He J., Ma M. (2011). Olfactory marker protein is critical for functional maturation of olfactory sensory neurons and development of mother preference. J. Neurosci..

[13] Youngentob S.L., Margolis F.L. (1999). OMP gene deletion causes an elevation in behavioral threshold sensitivity. Neuroreport.

[14] Nakashima A., Nakagawa T., Takano M., Nakashima N. (2020). Olfactory marker protein contributes to the evaluation of odour values by olfactory glomerular processing. Neurosci. Lett..

[15] Dibattista M., Reisert J. (2016). The Odorant receptor-dependent role of olfactory marker protein in olfactory receptor neurons. J. Neurosci..

[16] Reisert J., Yau K.-W., Margolis F.L. (2007). Olfactory marker protein modulates the cAMP kinetics of the odour-induced response in cilia of mouse olfactory receptor neurons. J. Physiol..

[17] Nakashima N., Nakashima K., Taura A., Takaku-Nakashima A., Ohmori H., Takano M. (2020). Olfactory marker protein directly buffers cAMP to avoid depolarization-induced silencing of olfactory receptor neurons. Nat. Commun..

[18] Nakashima N., Nakashima K., Taura A., Takaku A., Ohmori H., Takano M. (2018). Buffering cAMP in olfactory receptor neurons. J. Physiol. Sci..

[19] Kwon H.J., Koo J.H., Zufall F., Leinders-Zufall T., Margolis F.L. (2009). Ca^2+^ extrusion by NCX is compromised in olfactory sensory neurons of OMP^-/-^ mice. PloS One.

[20] Albeanu D.F., Provost A.C., Agarwal P., Soucy E.R., Zak J.D., Murthy V.N. (2018). Olfactory marker protein (OMP) regulates formation and refinement of the olfactory glomerular map. Nat. Commun..

[21] John J.A. St, Key B. (2005). Olfactory marker protein modulates primary olfactory axon overshooting in the olfactory bulb. J. Comp. Neurol..

[22] Imai T., Suzuki M., Sakano H. (2006). Odorant receptor-derived cAMP signals direct axonal targeting. Science.

[23] Nakashima N., Ishii T.M., Bessho Y., Kageyama R., Ohmori H. (2013). Hyperpolarisation-activated cyclic nucleotide-gated channels regulate the spontaneous firing rate of olfactory receptor neurons and affect glomerular formation in mice. J. Physiol..

[24] Nakashima A., Takeuchi H., Imai T., Saito H., Kiyonari H., Abe T., Chen M., Weinstein L.S., Yu C.R., Storm D.R., Nishizumi H., Sakano H. (2013). Agonist-independent GPCR activity regulates anterior-posterior targeting of olfactory sensory neurons. Cell.

[25] Nakashima N., Nakashima K., Nakayama T., Takaku A., Kanamori R. (2017). Dual expression of constitutively active Gα_s_-protein-coupled receptors differentially establishes the resting activity of the cAMP-gated HCN2 channel in a single compartment. Biochem. Biophys. Res. Commun..

[26] Nakashima N., Nakashima K., Nakashima A., Takano M. (2020). Olfactory marker protein captures cAMP produced via Gαs-protein-coupled receptor activation. Biochem. Biophys. Res. Commun..

[27] Nakashima N., Nakashima K., Nakashima A., Takano M. (2020). Olfactory marker protein elevates basal cAMP concentration. Biochem. Biophys. Res. Commun..

[28] Behrens M., Margolis J.W., Margolis F.L. (2003). Identification of members of the *Bex* gene family as olfactory marker protein (OMP) binding partners. J. Neurochem..

[29] Koo J.H., Gill S., Pannell L.K., Menco B.P.M., Margolis J.W., Margolis F.L. (2004). The interaction of Bex and OMP reveals a dimer of OMP with a short half-life. J. Neurochem..

[30] Carr V.M., Walters E., Margolis F.L., Farbman A.I. (1998). An enhanced olfactory marker protein immunoreactivity in individual olfactory receptor neurons following olfactory bulbectomy may be related to increased neurogenesis. J. Neurobiol..

[31] Wright N.T., Margolis J.W., Margolis F.L., Weber D.J. (2005). Refinement of the solution structure of rat olfactory marker protein (OMP). J. Biomol. NMR.

[32] Sanner M.F. (1999). Python: a programming language for software integration and development. J. Mol. Graph. Model..

[33] O’Boyle N.M., Banck M., James C.A., Morley C., Vandermeersch T., Hutchison G.R. (2011). Open Babel: an open chemical toolbox. J. Cheminf..

[34] Trott O., Olson A.J. (2010). AutoDock Vina: improving the speed and accuracy of docking with a new scoring function, efficient optimization, and multithreading. J. Comput. Chem..

[35] Stoddart L.A., Johnstone E.K.M., Wheal A.J., Goulding J., Robers M.B., Machleidt T., V Wood K., Hill S.J., Pfleger K.D.G. (2015). Application of BRET to monitor ligand binding to GPCRs. Nat. Methods.

[36] Smith P.C., Firestein S., Hunt J.F. (2002). The crystal structure of the olfactory marker protein at 2.3 Å resolution. J. Mol. Biol..

[37] Erecińska M., Silver I.A. (1994). Ions and energy in mammalian brain. Prog. Neurobiol..

[38] Menco B.P.M., Bruch R.C., Dau B., Danho W. (1992). Ultrastructural localization of olfactory transduction components: the G protein subunit G_olfα_ and type III adenylyl cyclase. Neuron.

[39] Suzuki H., Nikaido M., Hagino-Yamagishi K., Okada N. (2015). Distinct functions of two olfactory marker protein genes derived from teleost-specific whole genome duplication. BMC Evol. Biol..

[40] Berman H.M., Ten Eyck L.F., Goodsell D.S., Haste N.M., Kornev A., Taylor S.S. (2005). The cAMP binding domain: an ancient signaling module. Proc. Natl. Acad. Sci. U. S. A..

[41] Wong W., Scott J.D. (2004). AKAP signalling complexes: focal points in space and time. Nat. Rev. Mol. Cell Biol..

[42] Uda S., Saito T.H., Kudo T., Kokaji T., Tsuchiya T., Kubota H., Komori Y., Ozaki Y., Kuroda S. (2013). Robustness and compensation of information transmission of signaling pathways. Science.

[43] Baker H., Grillo M., Margolis F.L. (1989). Biochemical and immunocytochemical characterization of olfactory marker protein in the rodent central nervous system. J. Comp. Neurol..

[44] Budanova E.N., Bystrova M.F. (2010). Immunohistochemical detection of olfactory marker protein in tissues with ectopic expression of olfactory receptor genes. Biochem. (Mosc.) Suppl. Ser. A: Membr. Cell Biol..

[45] Kang N., Kim H., Jae Y., Lee N., Ku C.R., Margolis F., Lee E.J., Bahk Y.Y., Kim M.S., Koo J. (2015). Olfactory marker protein expression is an indicator of olfactory receptor-associated events in non-olfactory tissues. PloS One.

[46] Pronin A., Levay K., Velmeshev D., Faghihi M., Shestopalov V.I., Slepak V.Z. (2014). Expression of olfactory signaling genes in the eye. PloS One.

[47] Uhlén M., Fagerberg L., Hallström B.M., Lindskog C., Oksvold P., Mardinoglu A., Sivertsson Å., Kampf C., Sjöstedt E., Asplund A., Olsson I., Edlund K., Lundberg E., Navani S., Szigyarto C.A.-K., Odeberg J., Djureinovic D., Takanen J.O., Hober S., Alm T., Edqvist P.-H., Berling H., Tegel H., Mulder J., Rockberg J., Nilsson P., Schwenk J.M., Hamsten M., von Feilitzen K., Forsberg M., Persson L., Johansson F., Zwahlen M., von Heijne G., Nielsen J., Pontén F. (2015). Proteomics. Tissue-based map of the human proteome. Science.

[48] Beidler L.M., Smallman R.L. (1965). Renewal of cells within taste buds. J. Cell Biol..

[49] Bernier P.J., Bédard A., Vinet J., Lévesque M., Parent A. (2002). Newly generated neurons in the amygdala and adjoining cortex of adult primates. Proc. Natl. Acad. Sci. U.S.A..

[50] Bramhall N.F., Shi F., Arnold K., Hochedlinger K., Edge A.S.B. (2014). *Lgr5*-positive supporting cells generate new hair cells in the postnatal cochlea. Stem Cell Reports.

[51] Farbman A.I., Margolis F.L. (1980). Olfactory marker protein during ontogeny: immunohistochemical localization. Dev. Biol..

[52] Gould E., Reeves A.J., Graziano M.S.A., Gross C.G. (1999). Neurogenesis in the neocortex of adult primates. Science.

[53] Graziadei P.P., Graziadei G.A.M. (1980). Neurogenesis and neuron regeneration in the olfactory system of mammals. III. Deafferentation and reinnervation of the olfactory bulb following section of the fila olfactoria in rat. J. Neurocytol..

[54] Huang L.Y., DeVries G.J., Bittman E.L. (1998). Photoperiod regulates neuronal bromodeoxyuridine labeling in the brain of a seasonally breeding mammal. J. Neurobiol..

[55] Jhaveri D.J., Tedoldi A., Hunt S., Sullivan R., Watts N.R., Power J.M., Bartlett P.F., Sah P. (2018). Evidence for newly generated interneurons in the basolateral amygdala of adult mice. Mol. Psychiatr..

[56] Kaplan M.S., Hinds J.W. (1977). Neurogenesis in the adult rat: electron microscopic analysis of light radioautographs. Science.

[57] Yamasoba T., Kondo K. (2006). Supporting cell proliferation after hair cell injury in mature Guinea pig cochlea in vivo. Cell Tissue Res..

[58] Zhao M., Momma S., Delfani K., Carlén M., Cassidy R.M., Johansson C.B., Brismar H., Shupliakov O., Frisén J., Janson A.M. (2003). Evidence for neurogenesis in the adult mammalian substantia nigra. Proc. Natl. Acad. Sci. U.S.A..

[59] Koo J.H., Saraswati M., Margolis F.L. (2005). Immunolocalization of Bex protein in the mouse brain and olfactory system. J. Comp. Neurol..

[60] Zhang Y., Kim M.S., Jia B., Yan J., Zuniga-Hertz J.P., Han C., Cai D. (2017). Hypothalamic stem cells control ageing speed partly through exosomal miRNAs. Nature.

[61] Paul A., Chaker Z., Doetsch F. (2017). Hypothalamic regulation of regionally distinct adult neural stem cells and neurogenesis. Science.

